# Polyvascular atherosclerosis and renal dysfunction increase the odds of cognitive impairment in vascular disease: findings of the LipidCardio study

**DOI:** 10.1186/s40001-024-01734-6

**Published:** 2024-02-22

**Authors:** Maximilian König, Katie Palmer, Carolin Malsch, Elisabeth Steinhagen-Thiessen, Ilja Demuth

**Affiliations:** 1 Department of Internal Medicine D-Geriatrics, Universitätsmedizin Greifswald, Walther-Rathenau-Str. 49, 17475 Greifswald, Mecklenburg-Vorpommern Germany; 2 Department of Endocrinology and Metabolic Diseases (including Division of Lipid Metabolism), Charité-Universitätsmedizin Berlin, Corporate Member of Freie Universität Berlin and Humboldt-Universität Zu Berlin, Berlin, Germany; 3 Department of Clinical Geriatrics, NVS, Karolinska Institutet, Stockholm, Sweden; 4 Institute for Mathematics and Computer Science, University of Greifswald, Greifswald, Germany; 5 BCRT-Berlin Institute of Health Center for Regenerative Therapies, Berlin Institute of Health at Charité-Universitätsmedizin Berlin, Berlin, Germany

**Keywords:** CKD, PolyVD, Cognition, Cognitive impairment

## Abstract

**Introduction:**

Growing evidence suggests a causal role for atherosclerotic vascular disease in cognitive impairment and dementia. Atherosclerosis may present as monovascular disease (monoVD) or as widespread polyvascular atherosclerotic disease (polyVD). Evidence on the relationship between monoVD or polyVD and cognitive impairment is limited.

**Methods:**

We conducted a cross-sectional analysis of baseline data from the LipidCardio Study. The main outcome measure was the presence of cognitive impairment, defined as a Mini-Mental State Examination (MMSE) score < 26.

**Results:**

The mean age was 71.5 years, 30.3% were female, 17.3% had no evidence of large-vessel atherosclerosis, 71.1% had monoVD, and 11.7% had polyVD, defined as the presence of atherosclerosis in ≥ 2 vascular territories (coronary, cerebral, aortic, or lower extremity). A total of 21.6% had cognitive impairment according to the prespecified cutoff (MMSE < 26). Overall, the odds of cognitive impairment increased for each additional vascular territory affected by atherosclerosis [adjusted odds ratio 1.76, 95% confidence interval (CI) 1.21–2.57, *p* = 0.003]. Furthermore, there was evidence for an interaction between vascular disease and chronic kidney disease (CKD). The odds of cognitive impairment were not greater in the monoVD subgroup compared to those without any atherosclerosis, if CKD was absent (OR 0.98, 95% CI 0.48–2.10; *p* = 0.095), while the odds ratio (OR) of cognitive impairment with polyVD compared to no atherosclerosis was 2.71 (95% CI 1.10–6.92; *p* = 0.031). In contrast, in patients with CKD, both monoVD and polyVD were associated with significantly higher odds of cognitive impairment than no atherosclerosis.

**Conclusions:**

PolyVD is associated with increased odds of cognitive impairment. MonoVD is associated with cognitive impairment only in the presence of CKD.

**Supplementary Information:**

The online version contains supplementary material available at 10.1186/s40001-024-01734-6.

## Introduction

Atherosclerosis is a systemic disorder that principally affects large and medium-sized arteries throughout the arterial tree [1]. Common sites and manifestations of atherosclerosis include coronary artery disease (CAD), peripheral artery disease (PAD), aortic atherosclerotic disease, and cerebrovascular atherosclerotic disease (CVAD), i.e., atherosclerosis of intracerebral vessels and the arteries that supply the brain [1]. Atherosclerosis may present as localized disease limited to one vascular territory but also as widespread, diffuse atherosclerotic disease involving multiple vascular territories [2]. The European Society of Cardiology (ESC) guidelines define polyvascular disease (polyVD) as the “simultaneous presence of clinically relevant atherosclerotic lesions in at least two major vascular territories” [3].

Approximately 15 to 43% of patients with atherosclerosis present with polyVD, i.e., atherosclerotic disease in multiple vascular territories [4]. Importantly, polyVD is associated with poor outcomes [5, 6]; patients with polyVD experience significantly greater rates of major adverse cardiovascular events than individuals with monovascular atherosclerotic disease (monoVD) [7, 8].

The implications of atherosclerotic vascular disease go beyond the cardiovascular system. A steadily growing body of evidence suggests that atherosclerotic vascular disease of various locations, e.g., CAD, is associated with an increased risk of cognitive decline and dementia [9–11]. While there is consensus as to the causal role of cerebrovascular atherosclerosis in vascular cognitive impairment, vascular dementia, and mixed dementia [12], recent evidence suggests that vascular disease plays an important role in any form of dementia, including Alzheimer disease [13]. Furthermore, although there is growing evidence showing that atherosclerotic vascular disease in different vascular territories—including those of CAD and PAD—is associated with cognitive impairment [14], it is still not established whether atherosclerosis per se—irrespective of the vascular bed affected—is related to cognitive impairment and dementia and whether there is an exposure‒response relationship [15, 16].

Furthermore, it is unclear which pathways underlie the association between atherosclerotic vascular disease and cognitive impairment, although small vessel disease seems to be an important mediating factor between vascular disease and cognitive impairment [17]. The role of chronic kidney disease (CKD) has recently been investigated [18]. CKD, defined as a sustained decrease in the glomerular filtration rate < 60 ml/min/1.73 m^2^ or albuminuria ≥ 30 mg/d, is a risk factor for vascular disease and is associated with an increase in atherosclerotic burden [19]. Moreover, there is evidence that atherosclerosis in CKD is a generalized, systemic process that also involves small vessels [20]. Indeed, polyVD is frequently observed in CKD patients. [21] Likewise, the prevalence of mild cognitive impairment has been estimated to be as high as 30% to 63% in individuals with CKD [22].

Understanding the relationship between vascular disease and cognitive decline is important for identifying potentially modifiable therapeutic targets and implementing strategies to prevent or delay cognitive decline and dementia. The objective of the present study was to examine the association between the atherosclerotic burden, i.e., the number of vascular beds affected by atherosclerosis, and cognitive impairment. Specifically, we hypothesized that the odds of cognitive impairment increase with an increasing number of vascular beds involved. Furthermore, we aimed to explore whether the association differed depending on the presence of CKD.

## Methods

### Study population and design

We performed a cross-sectional analysis of baseline data from the LipidCardio study, which was conducted at a single tertiary academic cardiology center in Berlin, Germany, during 2016–2018 (study period of 17 months) and included 1005 consecutive patients (mean age at enrollment 70.9 ± 11.1 years, 70% male) who had been referred for cardiac catheterization [23]. These included patients with (a) suspected CAD, (b) asymptomatic and symptomatic patients with known CAD and suspected disease progression, or (c) patients with multivessel disease and recent incomplete revascularization. Patients with troponin-positive acute coronary syndrome and patients unable to provide informed consent were excluded [24].

### Ethics approval

The LipidCardio study was approved by the ethics committee at Charité-Universitätsmedizin Berlin (approval number: EA1/135/16). All participants provided written informed consent at the time of enrollment. The study was conducted in compliance with the principles of the Declaration of Helsinki.

### Cognitive functioning

Global cognitive functioning was assessed with the Mini-Mental State Examination (MMSE) in German [25], which assesses several areas of cognitive function: orientation, attention, memory, language, and visual construction. A total possible score between 0 and 30 was given, with lower scores indicating more impaired cognition [26]. The participants were categorized according to a prespecified threshold of 26 into “normal cognition” (MMSE score ≥ 26) or “cognitive impairment” (MMSE score < 26). The cutoff of 26 was chosen based on a critical review of previous studies [27–31]. We also used the MMSE score as a continuous variable in the analyses.

### Ascertainment of atherosclerosis manifestations

As the LipidCardio participants were recruited from among patients referred for cardiac catheterization, each participant had an up-to-date angiogram, which either confirmed the presence of coronary atherosclerosis or excluded it. The CAD diagnosis was made by the interventional cardiologist, who performed the coronary angiography; otherwise, he/she was not involved in the design, conduct or evaluation of the study. Information on the presence of non-coronary atherosclerosis (peripheral arterial disease, atherosclerotic aortic disease, or CVAD) was extracted from the patients’ electronic medical records.

### Covariables

Potential confounders and effect modifiers were identified a priori based on subject knowledge and a literature review. Preexisting diagnoses (diabetes mellitus type 2, hypertension) were extracted from the patients’ electronic medical records. Blood specimens were collected from all participants during cardiac catheterization, and selected laboratory values, including serum creatinine levels, were determined in an accredited central laboratory. The eGFR was estimated using the Chronic Kidney Disease Epidemiology Collaboration (CKD-EPI) equation based on the serum creatinine concentration, age and sex [32]. CKD was defined as an estimated glomerular filtration rate (eGFR) < 60 ml/min/1.73 m^2^. DNA was isolated from EDTA blood samples using the sbeadex livestock kit (LGC Genomics GmbH, Germany), which had been frozen at − 80 °C. Using KASP chemistry (LGC, Hoddesdon, UK), we genotyped the single nucleotide polymorphisms (SNPs) rs429358 and rs7412, which constitute the three isoforms of apolipoprotein E *APO* e2, *APO* e3, and *APO* e4. The *APO* e4 genotype was employed as a binary variable (*APO* e4 allele carrier status: 0 or ≥ 1 allele) [24]. For some participants (*n* = 63), DNA was not available. Body mass index (BMI) was calculated as weight in kilograms divided by the square of the size in meters and categorized into < 25 kg/m^2^ (normal), 25–30 kg/m^2^ (overweight), and > 30 kg/m^2^ (obese). Information on the current smoking status was collected via structured interviews by the study staff and coded as yes/no. Four age strata were used (< 60 years, 60–69 years, 70–79 years, ≥ 80 years).

### Statistics

The characteristics of participants with missing data and participants without missing data were compared using the *t* test, the Wilcoxon rank sum test, or the *χ*^2^ test.

We compared baseline participant characteristics according to vascular bed involvement (atherosclerosis in 0, 1, ≥ 2 vascular territories) using one-way analysis of variance (ANOVA) or the Kruskal‒Wallis test for continuous variables and *χ*^2^ tests for categorical variables.

Potential confounders and effect modifiers were identified a priori based on the relevant literature (diabetes mellitus type 2, CKD, *APO* e4 allele carrier status, hypertension, sex, BMI, current smoking status, and age).

Associations of all covariables with both the outcome of interest (cognitive impairment) and the main exposure of interest (number of vascular beds with atherosclerosis or PolyVD) were calculated using cross-tabulations and crude odds ratios. The *χ*^2^ test of independence was used to assess statistical significance.

We used logistic regression to calculate the crude odds ratio (OR) of cognitive impairment for a one-unit increase in vascular bed involvement. Next, all covariables were tested for confounding and effect modification by calculating stratum-specific estimates for each level of the potential confounder. We used multivariable logistic regression to quantify the odds of cognitive impairment resulting from an increasing number of vascular beds affected by atherosclerosis (no atherosclerosis, MonoVD, or PolyVD) and the odds ratios of monoVD and polyVD compared to participants who had zero vascular beds affected. In view of the small sample sizes in the stratified analyses and to confirm the results with a second regression method, we used Firth’s logistic regression [Heinze G, Ploner M, Jiricka L, Steiner G (2023). logistf: Firth’s Bias-Reduced Logistic Regression. R package version 1.26.0, https://CRAN.R-project.org/package=logistf]. All multivariable models were adjusted for age, sex, smoking status, diabetes mellitus type 2, hypertension, BMI, and *APO* e4 carrier status. CKD was also included in all final models that were not stratified according to CKD status. We further tested for effect modification by CKD by producing a model with interaction. The likelihood ratio test was used to test for evidence of interaction between vascular disease and CKD, and stratum-specific odds ratios (ORs) and 95% confidence intervals (CIs) were calculated for individuals with and without CKD.

### Sample size calculations

A priori sample size calculations with varying assumptions showed that with the given fixed sample size (*n* = 829), the study had a reasonable chance of detecting an important effect.

All analyses were performed using Stata/IC 15.0 for Mac (StataCorp, College Station, TX) or R Statistical Software (v4.2.2; R Core Team 2022).

### STROBE statement

The manuscript was prepared in compliance with the STrengthening the Reporting of Observational Studies in Epidemiology (STROBE) statement.

## Results

After excluding 176 of the 1005 participants enrolled in the LipidCardio study for whom no cognitive data were available, 829 observations were included in the present analysis. (Additional file 1: Figure S1).

Overall, the mean age of the participants in the analyzed sample (*n* = 829) was 71.5 years, 30.3% were female, 27.9% had diabetes, 29.8% had CKD, 80.7% had hypertension, and 68.4% were either overweight or obese. The prevalence of cognitive impairment according to the chosen cutoff (MMSE < 26) was 21.6%.

A total of 589 participants (71.1%) had evidence of atherosclerosis in one vascular territory (monovascular disease), 91 participants (11%) had evidence of atherosclerosis in two vascular territories, and 6 participants (0.7%) had evidence of atherosclerosis in three vascular territories. A total of 11.7% had polyvascular disease (polyVD), i.e., evidence of atherosclerosis in ≥ 2 vascular territories. No clinically recognized atherosclerotic vascular disease was present in 143 participants (17.3%). Table 1 summarizes the demographic and clinical characteristics of the participants according to the number of vascular beds affected by atherosclerosis. Overall, participants with missing data were comparable to those without missing data (Additional file 1: Table S1).

**Table 1:** Baseline characteristics according to the number of vascular beds with recognized atherosclerosis

Total *n* = 829	No atherosclerosis *n* = 143 (17.3)	Monovascular disease *n* = 589 (71.1)	Polyvascular disease *n* = 97 (11.7)	Missings
Age, years	65.8 ± 13.3	72.3 ± 10.0	74.7 ± 9.1	0
Age groups				0
< 60 years (*n* = 128)	49 (38.0)	73 (56.6)	7 (5.4)	
60–69 years (*n* = 201)	30 (14.9)	150 (74.6)	21 (10.5)	
70–79 years (*n *= 309)	42 (13.6)	225 (72.8)	42 (13.6)	
≥ 80 years (*n* = 190)	22 (11.6)	141 (74.2)	27 (14.2)	
Sex, male	62 (43.4)	440 (74.7)	76 (78.4)	0
MMSE (mean)	27.9 ± 2.5	27.0 ± 2.9	26.2 ± 2.7	0
MMSE (median)	29 (27–29)	28 (26–29)	27 (25–28)	0
MMSE < 26	15 (10.5)	130 (22.1)	34 (35.1)	0
*APO* e4 carrier	36 (27.1)	126 (23.1)	30 (34.5)	63
Diabetes mellitus Type 2	25 (17.5)	163 (27.7)	43 (44.3)	0
Hypertension	97 (67.8)	494 (83.9)	78 (80.4)	0
CKD (eGFR < 60 ml/min/1.73^2^)	27 (20.0)	169 (29.5)	43 (45.3)	26
Smoking	28 (19.7)	96 (16.4)	28 (28.9)	4
BMI > 25 kg/m^2^	55 (38.7)	248 (42.5)	43 (44.3)	6
BMI > 30 kg/m^2^	38 (26.8)	158 (27.1)	21 (21.7)	6

Data are presented as mean ± standard deviation, numbers(proportions), or median(25th-75th percentile)

*BMI* body mass index, *CKD* chronic kidney disease, *eGFR* estimated glomerular filtration rate, *MMSE* Mini-Mental State Examination;

There was clear evidence of a trend toward decreasing MMSE scores with increasing numbers of affected vascular beds (*p* for trend < 0.001). The mean MMSE score decreased by 0.82 (95% CI 0.46–1.18; *p* < 0.001) per increase in the number of affected vascular beds.

The prevalence of cognitive impairment (MMSE < 26) also increased gradually: 10.5% in people without signs of atherosclerosis, 22.1% in people with monoVD and 35.1% in people with polyVD (*p* for trend < 0.001). Participants without evidence of atherosclerosis were younger and more likely to be female (56.6%), whereas participants with polyVD more often had CKD (prevalence 45.3%) and diabetes and were more likely to be current smokers than participants with monoVD or without evident atherosclerosis (Table 1). BMI categories and *APO* e4 carrier status were distributed similarly across the groups.

The crude odds of cognitive impairment were 2.42 times greater in participants with monoVD than in participants without atherosclerotic disease [unadjusted odds ratio (OR) 2.42, 95% confidence interval (CI) 1.37–4.27, *p* = 0.002] and 4.61 times greater in participants with polyVD than in participants without evidence of atherosclerosis (unadjusted OR 4.61 95% CI 2.34–9.07, *p* < 0.001; Table 2).

**Table 2:** Characteristics of participants and associations with cognitive impairment (defined as MMSE score < 26)

Characteristic	Category	Odds ratio (95% confidence interval)	*p*-value
Sex	Female	1 (ref.)	–
	Male	1.12 (0.77–1.61)	0.557
Atherosclerosis manifestations	No atherosclerosis	1 (ref.)	–
	1 vascular bed	2.42 (1.37–4.27)	0.002
	≥ 2 vascular beds	4.61 (2.34–9.07)	< 0.001
Age group	< 60 years	1 (ref.)	–
	60–69 years	1.60 (0.74–3.47)	0.234
	70–79 years	3.39 (1.69–6.82)	0.001
	≥ 80 years	7.87 (3.88–15.97)	< 0.001
BMI, kg/m^2^	< 25	1 (ref.)	–
	25–30	0.80 (0.54–1.19)	0.277
	> 30	1.10 (0.72–1.68)	0.657
Diabetes mellitus Type 2	No	1 (ref.)	–
	Yes	1.83 (1.29–2.60)	0.001
Hypertension	No	1 (ref.)	–
	Yes	1.53 (0.97–2.41)	0.069
CKD	No	1 (ref.)	–
	Yes	2.45 (1.73–3.47)	< 0.001
Current smoking	No	1 (ref.)	–
	Yes	0.79 (0.53–1.23)	0.296
*APO* e4 carrier	No	1 (ref.)	–
	Yes	0.82 (0.55–1.24)	0.352

*BMI* body mass index, *CKD* chronic kidney disease, *MMSE* Mini-Mental State Examination, *ref.* reference category

The odds of cognitive impairment for a unit increase in the number of vascular bed was 2.10 (95% CI 1.52–2.91, *p* < 0.001; likelihood ratio test for departure from linear trend *p* = 0.549; Additional file 1: Table S3).

Table 2 and Additional file 1: Table S2 show the associations of variables with cognitive impairment and polyVD, respectively. The prevalence of cognitive impairment was similar in men and women (22.2% vs. 20.3%). There was strong evidence of a linear trend of increasing incidence of cognitive impairment with increasing age (< 60 years: 7.8%, 60–69 years: 11.9%, 70–79 years; 22.3%, ≥ 80 years: 40.0%, chi-square test of trend *p* < 0.001). Moreover, cognitive impairment was positively associated with an increasing number of atherosclerosis manifestations, type 2 diabetes mellitus, hypertension, and CKD (Table 2).

Conversely, univariate analyses provided evidence that polyVD was associated with cognitive impairment, type 2 diabetes mellitus, CKD, at least one *APO* e4 allele, and current smoking status (Additional file 1: Table S2).

While the crude analyses provided strong evidence of an association between an increasing number of vascular beds and evidence of atherosclerosis and cognitive impairment, there was evidence that the relationship between atherosclerotic burden and cognitive impairment was negatively and positively confounded by APO e4 carrier status, type 2 diabetes mellitus, age group, and CKD (Additional file 1: Table S3). There were also indications of effect modification by CKD (likelihood ratio test *p* = 0.045).

After adjusting for age group, sex, *APO* e4 carrier status, BMI, current smoking status, type 2 diabetes mellitus, hypertension, and CKD, there was still evidence of a 76% increase in the likelihood of cognitive impairment from no atherosclerosis to monoVD or polyVD (Table 3).

**Table 3:** Multivariable-adjusted* odds of cognitive impairment

	Adjusted odds ratio (95% confidence interval)	*p*-value
No atherosclerosis	1.0 (reference)	–
Monovascular disease	1.72 (0.90–3.29)	0.103
Polyvascular disease	3.08 (1.42–6.68)	0.005
Vascular beds (linear effect)	1.76 (1.21–2.57)	0.003

^*^Adjusted for age group, sex, *APO* e4 carrier status, body-mass index, current smoking, diabetes mellitus, hypertension, and chronic kidney disease; *N* = 733

We further tested the interaction between the number of vascular beds with atherosclerosis and CKD status in the multivariable-adjusted model, and the likelihood ratio test provided evidence against the null hypothesis of no effect modification by CKD (*p* = 0.030). Stratified by CKD and assuming a linear effect of the number of vascular beds with atherosclerosis and cognitive impairment, there was still evidence of increasing odds of cognitive impairment with an increasing number of affected vascular territories *in the absence of CKD* (linear effect OR 1.88, 95% CI 1.13–3.11, *p* = 0.015), while there was only very weak evidence of such a link in participants *with CKD* (linear effect OR 1.63, 95% CI 0.91–2.95, *p* = 0.102).

Next, we calculated separate parameter estimates comparing monoVD with no atherosclerosis and polyVD with no atherosclerosis. Table 4 and Additional file 1: Table S4 show the results of Firth’s logistic regression analyses, which we used due to the small sample sizes, with resulting wide confidence intervals especially in the CKD group (*n* = 214). Individuals without CKD were not found to have a higher likelihood of cognitive impairment in the presence of monoVD compared to those without atherosclerosis (OR 0.98, 95% CI 0.48–2.10; *p* = 0.095), while individuals with polyVD had approximately three times greater odds of cognitive impairment compared to those without atherosclerosis (OR 2.71, 95% CI 1.10–6.92; *p* = 0.031) or individuals with monoVD (OR 2.78, 95% CI 1.40–5.43; *p* = 0.004).

**Table 4:** Odds of cognitive impairment by number of vascular beds involved among individuals with and without chronic kidney disease (Firth’s logistic regression)

	Adjusted* odds ratio (95% confidence interval)	*p*-value
CKD (*n* = 214)		
0 vascular beds	1.0 (reference)	–
1 vascular bed^#^	4.60 (1.35–23.98)	0.012
≥ 2 vascular beds^+^	4.24 (1.04–24.37)	0.043
No CKD (*n* = 520)		
0 vascular beds	1.0 (reference)	–
1 vascular bed^#^	0.98 (0.48–2.10)	0.095
≥ 2 vascular beds^+^	2.71 (1.10–6.92)	0.031

CKD, chronic kidney disease, ^#^ monovascular atherosclerotic disease, ^+^polyvascular atherosclerotic disease; *adjusted for age group, sex, *APO* e4 carrier status, body-mass index, current smoking, diabetes mellitus, hypertension

In contrast, participants with CKD were approximately five times more likely to experience cognitive impairment in both monoVD and polyVD compared to those without atherosclerosis, respectively (OR 4.60, 95% CI 1.35–23.98, *p* = 0.012; OR 4.24, 95% CI 1.04–24.37, *p* = 0.043; Table 4). Moreover, there was no evidence that polyVD was associated with increased odds of cognitive impairment compared to monoVD (OR 0.92, 95% CI 0.42–2.00, *p* = 0.838). However, it should be noted that the number of patients with CKD was small (*n* = 214), and the 95% confidence intervals were wide; i.e., the true effect could be rather small, or even significantly greater.

## Discussion

Our cross-sectional study suggested that clinically recognized atherosclerosis in any vascular bed is not associated with increased odds of cognitive impairment. Only individuals with either polyvascular disease or monovascular atherosclerosis plus CKD had increased odds of cognitive impairment compared to individuals without atherosclerosis. In addition to CKD, polyvascular disease was not associated with any further increase in the odds of cognitive impairment compared to monovascular disease with concomitant CKD.

These results confirm previous findings of others and extend beyond the established knowledge by highlighting the importance of additional vascular territory involvement and co-occurrence with CKD for the unfavorable association of atherosclerotic vascular disease with cognitive impairment [33, 34]. Numerous studies have shown an association between atherosclerotic vascular disease and cognitive impairment [14, 35]. In the Cardiovascular Health Study, Newman et al. reported that CVD, particularly CAD and PAD, increases the risk of dementia. Like in the present study, they found an increase in risk with increasing extent of vascular disease [36]. Similarly, a recent analysis of the Dallas Heart Study revealed a significant linear trend toward decreasing scores on the Montreal Cognitive Assessment Test, another commonly used cognitive screening test, as the number of atherosclerosis indicators increased [37]. The latter study was similar to the present study in that we considered multiple vascular territories (coronary atherosclerosis, abdominal aortic, peripheral, and cerebrovascular atherosclerosis), as they simultaneously examined different manifestations of atherosclerosis (coronary and aortic) in relation to global cognitive function in a study of middle-aged adults [38].

In contrast, a recent analysis of the Stabilization of Atherosclerotic Plaque by Initiation of Darapladib Therapy Trial (STABILITY) did not find evidence for an association between cognitive impairment and polyvascular disease or impaired kidney function [39]. A population-based study of older adults also revealed no independent association between CAD in general and cognitive impairment in noninstitutionalized, community-dwelling older adults. However, among men, they found that “complicated CAD” negatively affects cognitive functioning [40]. In the present study, almost all individuals with clinically recognized atherosclerosis manifestations had CAD, and only those with polyVD had an additional non-coronary manifestation, i.e., coronary plus non-coronary atherosclerosis. Thus, the results of the present study also support the hypothesis that CAD alone is not associated with cognitive impairment; however, there is evidence of such an association in the presence of concomitant non-coronary atherosclerosis or CKD. This finding is consistent with previous findings that non-coronary atherosclerosis is more consistently associated with cognitive impairment, while the evidence for an association between CAD and cognitive impairment is mixed, with some studies suggesting an association while others do not [15, 36, 37].

The differences between individuals with and without CKD are also of interest. In participants *without CKD*, i.e., with preserved kidney function, monoVD was not associated with increased odds of cognitive impairment compared to no atherosclerosis. However, in participants with polyVD compared with monoVD or no clinically recognized atherosclerotic disease, the odds of cognitive impairment were approximately two to threefold greater. In contrast, in participants *with impaired kidney function *(i.e., *CKD*), both monoVD and polyVD were associated with markedly increased odds of cognitive impairment compared to participants without atherosclerosis. This increased likelihood of cognitive impairment irrespective of the severity of atherosclerotic vascular disease is noteworthy. This may be explained by the fact that CKD is characterized by universal vascular damage and dysfunction, including of the small vessels, and an atherosclerotic burden that starts early and grows steadily [19, 21, 41]. Indeed, small vessel disease could be pivotal for the association between atherosclerosis and cognitive impairment [42, 43]. In this regard, CKD has been suggested to affect cognition partly via the high occurrence of cerebral small vessel disease [44]. Additionally, there is evidence that polyVD is significantly associated with microvascular dysfunction, and microvascular pathology may partly account for the high cardiovascular risk of individuals with polyVD [45].

Unfortunately, albuminuria was not available in the LipidCardio cohort. Albuminuria is not only a hallmark of kidney dysfunction, but also a marker of microvascular endothelial dysfunction. Studies, such as the one by Tanaka et al. have demonstrated a correlation between albuminuria and cerebral small vessel disease, indicating that increased urinary albumin excretion may be indicative of microvascular damage in the brain [46]. Additionally, (imaging) studies have underscored anatomical microvascular similarities between brains of dementia patients and kidneys of individuals with albuminuria, linking kidney disease and cerebral small vessel disease [47–49]. Furthermore, studies, including the one by Pereira et al. revealed subcortical cognitive impairment in dialysis patients, aligning with the impact of microvascular dysfunction on cognitive function [50].

Importantly, CKD is common in older adults and was also highly prevalent (30%) in our sample [45, 51, 52]. Among individuals with CKD, the prevalence of mild cognitive impairment has been estimated to be as high as 30% to 63% [22], but this cannot be fully explained by established vascular risk factors [53]. Increasing evidence suggests that cognitive impairment in CKD patients represents a clinical entity that is different in many ways from the cognitive impairment observed in individuals without CKD [22].

We found no evidence that the likelihood of cognitive impairment increases with the number of vascular beds affected by atherosclerosis in individuals with CKD. This could be explained by the fact that CKD as such is already associated with increased odds of cognitive impairment, which can be explained by a diffuse, generalized micro- and macrovascular dysfunction typical of CKD, being present, whether polyVD or monoVD has been detected.

In this sense, CKD and polyVD may both indicate generalized vascular involvement equally.

In addition to the abovementioned aspects, our study has several limitations and strengths. Due to the cross-sectional nature of the study, we cannot establish causality or the direction of causality. Contrary to the common presumption that atherosclerosis impacts cognitive performance, reverse causation is conceivable; for example, individuals with cognitive impairment may be less likely to receive guideline-based treatments for CV risk factors. Furthermore, people with cognitive impairment might be less likely to receive secondary prevention for CVD. Consequently, atherosclerosis may progress and become symptomatic (in a second or third vascular territory) and be more likely to be detected and documented. A further limitation is that the MMSE has ceiling effects [22, 35]. In the present study, the ceiling effect may have resulted in individuals with cognitive impairment undetected and misclassified as “normal” by the MMSE with this particular cutoff; these individuals may have been identified with another test or another cutoff [36].

Another limitation is that the identification of non-coronary atherosclerosis was based only on a review of the participants’ electronic medical records, since the medical records are likely to differ in quality and completeness. Nevertheless, we have no indication that differences in quality or completeness may not have been randomly distributed with respect to exposure or outcome.

Moreover, it was not possible to reasonably consider the role of lipids in the analyses because the participants’ history of exposure to lipid-lowering therapies was highly variable.

There was also potential for selection bias. The LipidCardio cohort included a sample of patients who had been referred for coronary angiography. Patients may have been referred for coronary angiography more likely if they had no cognitive impairment, while people with dementia or signs of cognitive impairment may have been less likely to be referred for invasive cardiac diagnostics [54]. Furthermore, those without evidence of atherosclerosis, despite having a negative coronary angiogram, were not necessarily healthy controls since they were likely to have another pathological condition, accounting for their thoracic pain or discomfort. This other condition may also be associated with cognitive impairment, which may have led to an underestimation of the association between atherosclerosis and cognitive impairment.

With respect to generalizability, the sample and the results are certainly not representative of the general population. We used consecutive sampling, which is a form of nonprobability sampling. Since, we could enroll a large proportion of all patients who underwent cardiac catheterization at this tertiary center during the study period, the findings may be generalized to the population cared for in this hospital and other comparable groups of patients.

A strength of the study is that the presence of coronary atherosclerosis was confirmed or excluded by coronary angiography for each participant in a comparably large cohort, i.e., an invasive diagnostic procedure, which represents the gold standard for CAD diagnosis.

### Clinical relevance

Findings from the present study suggest that widespread polyvascular atherosclerosis and vascular disease plus CKD are associated with increased odds of cognitive impairment. This may suggest promising approaches to preventing cognitive impairment, namely, focusing on vascular health and paying more attention to maintaining kidney function, especially given the limited success of other approaches. In the context of the literature, these results suggest that the diagnostic work-up of patients with atherosclerosis should include a systematic search for atherosclerosis manifestations in other vascular beds to identify individuals with polyVD since this additional knowledge can contribute to better risk stratification, given that polyvascular disease is associated with an increased risk of several adverse outcomes, including cognitive impairment. Furthermore, the importance of including a screening tool for cognitive impairment, e.g., as part of a comprehensive geriatric assessment integrated into the clinical management of atherosclerosis patients, is emphasized.

Given the projected worldwide increase in the number of people affected by atherosclerotic vascular disease and dementia, further insight into causal mechanisms or common pathways underlying the observed connection is needed. In addition, the results underline that CKD is a relevant, imminent threat to patients with vascular disease, not only in view of cardiovascular events but also cognitive impairment. Notably, CKD is often disregarded in everyday medical practice and needs additional attention.

## Conclusion

In conclusion, the present study shows that atherosclerotic vascular disease is associated with increased odds of cognitive impairment. However, this is only the case if atherosclerosis is present as polyvascular disease or is accompanied by CKD. Atherosclerosis limited to one vascular bed is not associated with cognitive impairment. Concomitant CKD and polyvascular disease may indicate generalized systemic atherosclerosis, where all vascular beds are likely to be similarly affected.

### Supplementary Information

**Additional file 1: Figure S1.** Flowchart of sample selection. **Table S1.** Characteristics of participants with and without missing data. **Table S2.** Characteristics of participants and associations with polyvascular disease (reference category: monovascular disease). **Table S3.** Crude and one-variable adjusted odds ratios of cognitive impairment for a one unit increase in vascular bed involvement. **Table S4.** Multivariable-adjusted* odds of cognitive impairment (Firth Regression).

## Data Availability

Groups interested in data or biobank samples/DNA from LipidCardio should contact the study coordinating PI Ilja Demuth at ilja.demuth@charite.de for the data-sharing application form. Each application will be reviewed by the LipidCardio PIs and the decision communicated to the applicants usually within 6 weeks of submission.
