# Supplementary material for: Polyvascular atherosclerosis and renal dysfunction increase the odds of cognitive impairment in vascular disease: findings of the LipidCardio study
Source: Eur J Med Res. 2024 Feb 22;29:141. doi: 10.1186/s40001-024-01734-6 (PMC10882759; doi:10.1186/s40001-024-01734-6)
Supplement: Supplementary file 1 — Additional file 1: Figure S1. Flowchart of sample selection. Table S1. Characteristics of participants with and without missing data. Table S2. Characteristics of participants and associations with polyvascular disease (reference category: monovascular disease). Table S3. Crude and one-variable adjusted odds ratios of cognitive impairment for a one unit increase in vascular bed involvement. Table S4. Multivariable-adjusted* odds of cognitive impairment (Firth Regression). [file 40001_2024_1734_MOESM1_ESM.docx]

**Supplementary Figures and Tables**

**Polyvascular atherosclerosis and renal dysfunction increase the odds of cognitive impairment in vascular disease: findings of the LipidCardio study**

Maximilian König^1,2^, Katie Palmer^3^, Carolin Malsch^4^, Elisabeth Steinhagen-Thiessen^2^, Ilja Demuth^2,5^

^1^Department of Internal Medicine D – Geriatrics, Universitätsmedizin Greifswald, Mecklenburg-Vorpommern, Germany

^2^Department of Endocrinology and Metabolic Diseases (including Division of Lipid Metabolism), Charité–Universitätsmedizin Berlin, corporate member of Freie Universität Berlin and Humboldt-Universität zu Berlin, Berlin, Germany

^3^Department of Clinical Geriatrics, NVS, Karolinska Institutet, Stockholm, Sweden

^4^Institute for Mathematics and Computer Science, University of Greifswald, Greifswald, Germany

^5^BCRT - Berlin Institute of Health Center for Regenerative Therapies, Berlin Institute of Health at Charité–Universitätsmedizin Berlin, Berlin, Germany

**Supplementary Figure 1.**

LipidCardio cohort n = 1005

Analysed Sample

n = 829

Without cognitive data

n = 176

Missing data:

*APO* e4 n=63

BMI n=6

CAD n=2

eGFR n=26

**Supplementary Figure 1.** Flow chart of sample selection. *APO* e4 = *APO* e4 carrier status; BMI = body mass index, CAD = coronary artery disease, eGFR = estimated glomerular filtration rate.

**Supplementary Table 1.** Characteristics of participants with and without missing data.

|  | **With missing data**  **N=733** | **Without missing data**  **N=96** | **p-value** |
| --- | --- | --- | --- |
| Age, years | 73,4±9.3 | 71.3±11.0 | 0.073 |
| Male sex | 60(62.5) | 518(70.1) | 0.101 |
| Overweight | 35(38.9) | 311(42.4) | 0.781 |
| Obese | 26(28.9) | 191(26.1) | 0.781 |
| MMSE score | 27.1±0.3 | 27.1±0.1 | 0.912 |
| Polyvascular disease | 12(12.5) | 85(11.6) | 0.560 |
| Monovascular disease | 64(66.7) | 525(71.6) | 0.560 |
| CKD | 26(37.1) | 213(39.1) | 0.158 |

*Notes and abbreviations:* Data are mean±standard deviation or numbers(percentages), Abbreviations: MMSE, Mini-Mental State Examination; CKD, chronic kidney disease.

**Supplementary Table 2.** Characteristics of participants and associations with polyvascular disease (reference category: monovascular disease)

| **Characteristic** | **Category** | **Odds ratio**  **(95% confidence interval)** | **p-value** |
| --- | --- | --- | --- |
| **Sex** | Female | 1(ref.) | - |
|  | Male | 1.23(0.73-2.06) | 0.441 |
| **Cognitive impairment** | No (MMSE >= 26) | 1 (ref.) | - |
|  | Yes (MMSE < 26) | 1.91 (1.20-3.02) | 0.006 |
| **Age group** | < 60 years | 1(ref.) | - |
|  | 60-69 years | 1.46 (0.59-3.59) | 0.410 |
|  | 70-79 years | 1.95(0.84-4.52) | 0.121 |
|  | ≥ 80 years | 2.00(0.83-4.80) | 0.123 |
| **BMI, kg/m^2^** | < 25 | 1(ref.) | - |
|  | 25-30 (overweight) | 0.94(0.57-1.53) | 0.790 |
|  | > 30 (obese) | 1.29(0.40-1.29) | 0.128 |
| **Diabetes mellitus type 2** | No | 1(ref.) | - |
|  | Yes | 2.08(1.34-3.23) | 0.001 |
| **Hypertension** | No | 1(ref.) | - |
|  | Yes | 0.79(0.46-1.36) | 0.397 |
| **CKD** | No | 1(ref.) | - |
|  | Yes | 1.98(1.27-3.08) | 0.003 |
| **Current smoking** | Yes | 2.07(1.27-3.38) | 0.004 |
|  | No | 1 (ref.) | - |
| ***APO* e4** | No | 1(ref.) | - |
|  | Yes | 1.75(1.10-2.85) | 0.023 |

*Notes and abbreviations:* BMI, body mass index; CKD, chronic kidney disease; *APO* e4, *APO* e4 carrier status, MMSE, Mini-Mental State Examination, ref., reference category.

**Supplementary Table 3.** Crude and one-variable adjusted odds ratios of cognitive impairment for a one unit increase in vascular bed involvement.

| **Adjustment** | **Odds ratio**  **(95% confidence interval)** | **P-value** | **P interaction** | **N** |
| --- | --- | --- | --- | --- |
| **None (crude)** | 2.10(1.52-2.91) | <0.001 | - | 829 |
| **Sex** | 2.13(1.53-2.95) | <0.001 | 0.641 | 829 |
| ***APO* e4** **carrier** | 2.23 (1.58-3.14) | <0.001 | 0.728 | 766 |
| **Hypertension** | 2.08(1.50-2.88) | <0.001 | 0.960 | 829 |
| **Diabetes mellitus type 2** | 1.97(1.42-2.73) | <0.001 | 0.455 | 829 |
| **Age group** | 1.81(1.29-2.54) | <0.001 | 0.961 | 829 |
| **Smoking** | 2.14(1.54-2.97) | <0.001 | 0.672 | 825 |
| **Chronic kidney disease** | 1.95(1.40-2.73) | <0.001 | 0.045 | 803 |
| **BMI categories** | 2.10(1.52-2.90) | <0.001 | 0.341 | 823 |

*Notes and abbreviations:* BMI, body mass index;

**Supplementary Table 4.** Multivariable-adjusted* odds of cognitive impairment (Firth Regression)

|  | **Adjusted Odds ratio**  **(95% confidence interval)** | **P-value** |
| --- | --- | --- |
| **No atherosclerosis** | 1.0 (reference) | - |
| **Monovascular disease** | 1.65 (0.86-3.16) | 0.14 |
| **Polyvascular disease** | 2.84(1.30-6.19) | 0.01 |
| **Vascular beds (linear effect)** | 1.69(1.16-2.48) | 0.01 |

*Notes and abbreviations:* *adjusted for age group, sex, *APO* e4 carrier status, body-mass index, current smoking, diabetes mellitus, hypertension, and chronic kidney disease; N= 734
